# In vivo transport of Gd-DTPA^2-^ into human meniscus and cartilage assessed with delayed gadolinium-enhanced MRI of cartilage (dGEMRIC)

**DOI:** 10.1186/1471-2474-15-226

**Published:** 2014-07-09

**Authors:** Ulf Sigurdsson, Carl Siversson, Eveliina Lammentausta, Jonas Svensson, Carl-Johan Tiderius, Leif E Dahlberg

**Affiliations:** 1 Department of Orthopaedics, Lund University, Skåne University Hospital, SE-205 02 Malmö, Sweden; 2 Medical Radiation Physics, Department of Clinical Sciences, Lund University, Skåne University Hospital, SE-205 02 Malmö, Sweden; 3 Department of Diagnostic Radiology, Oulu University Hospital, PO Box 50, FI-90029 OYS Oulu, Finland; 4 Medical Radiation Physics, Department of Clinical Sciences, Lund University, Skåne University Hospital, SE-205 02 Malmö, Sweden; 5 Department of Orthopaedics, Lund University, Skåne University Hospital, SE-221 85 Lund, Sweden; 6 Department of Orthopaedics, Clinical Sciences Lund, Lund University, Skåne University Hospital, SE-221 85 Lund, Sweden

**Keywords:** dGEMRIC, Glycosaminoglycans, Meniscus, Cartilage

## Abstract

**Background:**

Impaired stability is a risk factor in knee osteoarthritis (OA), where the whole joint and not only the joint cartilage is affected. The meniscus provides joint stability and is involved in the early pathological progress of OA. Delayed gadolinium-enhanced MRI of cartilage (dGEMRIC) has been used to identify pre-radiographic changes in the cartilage in OA, but has been used less commonly to examine the meniscus, and then using only a double dose of the contrast agent. The purpose of this study was to enable improved early OA diagnosis by investigate the temporal contrast agent distribution in the meniscus and femoral cartilage simultaneously, in healthy volunteers, using 3D dGEMRIC at two different doses of the contrast agent Gd-DTPA^2-^.

**Methods:**

The right knee in 12 asymptomatic volunteers was examined using a 3D Look-Locker sequence on two occasions after an intravenous injection of a double or triple dose of Gd-DTPA^2-^ (0.2 or 0.3 mmol/kg body weight). The relaxation time (T_1_) and relaxation rate (R_1_ = 1/T_1_) were measured in the meniscus and femoral cartilage before, and 60, 90, 120 and 180 minutes after injection, and the change in relaxation rate (ΔR_1_) was calculated. Paired t-test and Analysis of Variance (ANOVA) were used for statistical evaluation.

**Results:**

The triple dose yielded higher concentrations of Gd-DTPA^2-^ in the meniscus and cartilage than the double dose, but provided no additional information. The observed patterns of ΔR_1_ were similar for double and triple doses of the contrast agent. ΔR_1_ was higher in the meniscus than in femoral cartilage in the corresponding compartments at all time points after injection. ΔR_1_ increased until 90-180 minutes in both the cartilage and the meniscus (p < 0.05), and was lower in the medial than in the lateral meniscus at all time points (p < 0.05). A faster increase in ΔR_1_ was observed in the vascularized peripheral region of the posterior medial meniscus, than in the avascular central part of the posterior medial meniscus during the first 60 minutes (p < 0.05).

**Conclusion:**

It is feasible to examine undamaged meniscus and cartilage simultaneously using dGEMRIC, preferably 90 minutes after the injection of a double dose of Gd-DTPA^2-^ (0.2 mmol/kg body weight).

## Background

In knee osteoarthritis (OA) a substantial risk factor is impaired stability [1]. Several studies have demonstrated that the meniscus plays an important role in knee stability, in addition to providing congruity and the capacity to bear load [2-4]. Knee OA is traditionally diagnosed based on clinical symptoms and radiographic signs. Radiographic changes are late events in the process during which the menisci and cartilage gradually deteriorate [5,6]. It is therefore important to develop diagnostic methods that can identify these degenerative changes in the meniscus and cartilage matrix, to enable detection and treatment of OA before irreversible damage or loss of tissue has occurred. The medial meniscus has attracted particular interest, and patients with meniscus injury are commonly used as a model to study early events in the development of OA [7-9].

Meniscus and articular cartilage consist of 60-80% water and 20-40% organic matter [10,11]. The main components of the organic matter are collagen and proteoglycan (a core protein with glycosaminoglycans attached). The contents of collagen and proteoglycan differ in the meniscus and articular cartilage, and also between regions within these tissues. The peripheral, vascular one-third of the meniscus consists almost exclusively of type I collagen, (80% by dry weight) [12]. In the avascular, central two-thirds of the meniscus, 70% is collagen (by dry weight), of which 40% is type I and 60% is type II [12]. The proteoglycan content in the meniscus is about 15% (dry weight) [12,13]. In articular cartilage, type II collagen is predominant, with a content of about 50-60% (dry weight); the other main component being proteoglycan, which constitutes about 15-30% (dry weight) [14]. The glycosaminoglycan (GAG) concentration in the meniscus is only 0.3-0.8% (wet weight), compared with 2.0% (wet weight) in articular cartilage [13,15].

Changes in cartilage GAGs are often studied to identify early changes in the tissue matrix and to study the progression of OA. The methods used today to detect pre-radiographic OA include the assessment of biochemical markers in synovial fluid, serum, and urine, as well as new molecular imaging techniques by MRI, such as delayed gadolinium-enhanced MRI of cartilage (dGEMRIC), glycosaminoglycan chemical exchange saturation transfer (gagCEST) and sodium magnetic resonance imaging (Na-MRI) [16-22]. dGEMRIC has been used to estimate the depletion of GAG in articular cartilage during the early stages of OA in research and in the clinical setting [23-25]. In dGEMRIC, the fixed charge density of GAG is studied *in vivo* by quantitative measurements of the longitudinal relaxation time (T_1_) of articular cartilage in the presence of the ionic contrast agent Gd-DTPA^2-^ (T_1(Gd)_). Gd-DTPA^2-^ distribution, which is inversely related to the concentration of negatively charged GAGs in the cartilage, is suggested as a measure of cartilage quality [18,19]. Longer T_1_ values correspond to lower concentrations of Gd-DTPA^2-^, and hence higher cartilage quality.

The dGEMRIC technique has rarely been applied to the meniscus, and in the few studies published a dose of 0.2 mmol/kg of Gd-DTPA^2**-**^, a so-called double dose, has been used [26,27]. In the present study we have compared double and triple doses to investigate the feasibility of dGEMRIC in clinical meniscus diagnostics.

The specific aims of this study were to investigate:

1. whether a triple dose provided any addition information, not provided by the double dose,

2. whether there was a difference between the uptake of contrast agent in the meniscus and joint cartilage, regarding the amount and when after injection the maximum level was attained, and

3. whether there was a difference in the uptake of contrast agent within the meniscus and in the meniscus at different locations in the knee joint.

## Methods

### Subjects

Twelve asymptomatic healthy volunteers (5 males and 7 females), aged 23-28 years (mean 25 years), were included after diagnostic MRI, demonstrating no pathological changes. Before inclusion, the nature of the procedure was fully explained to all subjects and written informed consent was obtained. The exclusion criteria were: 1. history of injury or pain in the knee; 2. contraindications for MRI (i.e. metal prosthesis, claustrophobia, serious allergy to contrast agent); 3. abnormality at physical examination of the knee; 4. abnormality in renal function. The study was approved by the ethics review board at Regionala Etikprövningskommittéen, Lund, Sweden.

### MRI

Intravenously injected Gd-DTPA^2**-**^ (Magnevist®, Bayer Schering Pharma AG, Berlin, Germany) was used as contrast agent. The injection of Gd-DTPA^2-^ was given in an antecubital vein. Zero time was defined as the end of the injection. After injection, the subjects walked for ten minutes at a slow pace, to optimize the distribution of Gd-DTPA^2-^ in the meniscus and cartilage.

Each subject was examined using both double (0.2 mmol/kg body weight) and triple (0.3 mmol/kg body weight) doses, administered on two different occasions separated by 5-6 months. Each subject was randomized to receiving the double or triple dose at the first examination. All imaging was performed on a Siemens Magnetom Sonata 1.5 T scanner with a CP extremity coil (Siemens Healthcare, Erlangen, Germany). Identical MRI protocols were used on both occasions. Each examination included quantitative T_1_ measurements which were performed before and 60, 90, 120 and 180 min after the injection of the contrast agent. A 3D Look-Locker sequence (FOV 160 × 160 × 90 mm, matrix 256 × 256 × 30 pixels, TR 2500 ms, flip angle 6°, 10 TIs), was used to acquire the 3D T_1_ maps. The acquisition time was 10 minutes and 42 seconds. T_1_ was calculated using the pre-calculated flip angle correction method, and the associated flip angle slice profile was acquired from previous phantom measurements [28]. All data were evaluated using software programmed in MATLAB (The MathWorks Inc., Natick, MA, USA).

### Evaluation of MRI measurements

T_1_ was measured before (T_1pre_) and four times after (T_1(Gd)_) contrast agent injection (60, 90, 120 and 180 minutes). From these T_1_ values, the change in relaxation rate, ΔR_1_, was calculated according to: ΔR_1_ = 1/T_1(Gd)_-1/T_1pre_), which reflects the post injection concentration of Gd-DTPA^2-^ in the tissue. The primary advantage of using 3D rather than 2D measurements in this study was that it was possible to choose the exact slice on which the regions of interest (ROIs) are defined after image acquisition.

**Figure 1:**
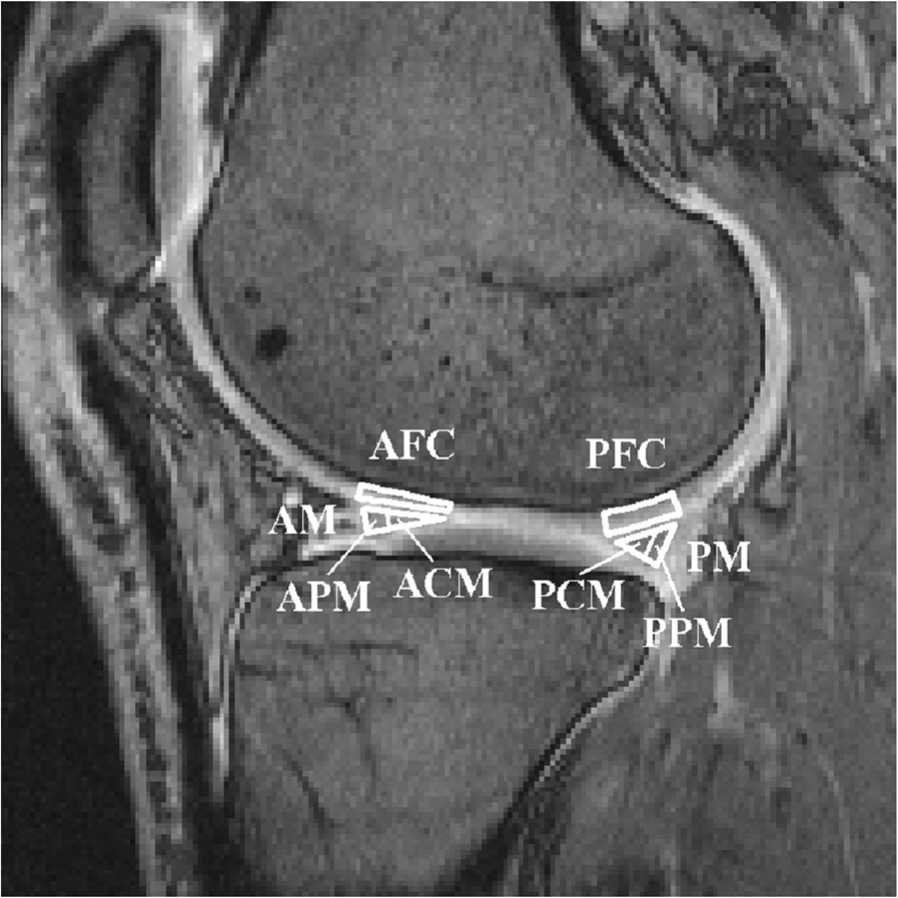
**Sagittal view of a knee joint.** The average value of T_1_ was calculated in the following regions of interest: AFC = Anterior Femoral Cartilage, PFC = Posterior Femoral Cartilage, AM = Anterior Meniscus, ACM = Anterior Central Meniscus (inner two-thirds), APM = Anterior Peripheral Meniscus (outer one-third), PM = Posterior Meniscus, PCM = Posterior Central Meniscus (inner two-thirds) and PPM = Posterior Peripheral Meniscus (outer one-third).

Two sagittal slices, one in the lateral and one in the medial compartment, were selected from the 3D volume to enable analysis of the weight-bearing parts of the meniscus and femoral cartilage. ROIs were drawn to cover the lateral and medial anterior and posterior regions of the meniscus, and the lateral and medial anterior and posterior femoral cartilage (Figure 1), in accordance with a scheme partly derived from Eckstein et al. [29]. The value of T_1_ for each ROI was calculated. The meniscus was also divided into a peripheral vascular region (the outer one-third of the meniscus) and a central avascular region (the inner two-thirds of the meniscus) to enable calculations of T_1_ in these parts of the meniscus (Figure 1). All ROIs were drawn by a single investigator. The paired t-test and analysis of variance (ANOVA) were used for statistical evaluation. The results are presented in the figures as mean values and 95% confidence interval (95% CI). A p-value <0.05 was considered to indicate statistical significance.

## Results

### Relaxation time of the meniscus and femoral cartilage before contrast agent injection

A difference was found in the average value of T_1pre_ between the meniscus and femoral cartilage. Similar values of T_1pre_ were seen in each tissue on the two measurement occasions. The mean values of T_1pre_ over the four compartments of the meniscus using the double dose of contrast agent were 617 ± 67 and 624 ± 65 ms, on occasions one and two, respectively. The corresponding mean values of T_1pre_ in the articular cartilage were 662 ± 75 ms and 655 ± 57 ms.

### Effect of contrast agent dose on meniscus image enhancement

**Figure 2:**
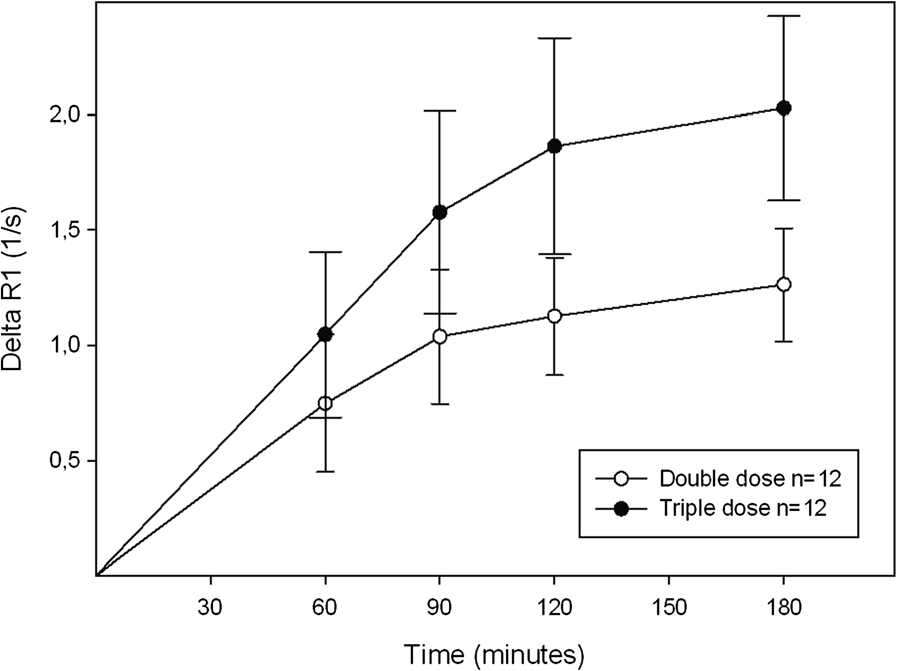
**Change in relaxation rate (ΔR**_**1**_ **± SD) in the posterior medial meniscus, after injection of a double (ο) or a triple (•) dose of Gd-DTPA**^**2- **^**(0.2 mmol/kg and 0.3 mmol/kg body weight, respectively).** Significantly higher mean values were observed after injection of the triple dose (ANOVA, p < 0.001).

The uptake of contrast agent was clearly visible in the meniscus after both double and triple doses. The triple dose resulted in higher values of ΔR_1_ than the double dose in all four compartments of the meniscus, as exemplified by the posterior medial compartment in Figure 2. It can also be seen from this figure that ΔR_1_ increased until 90 minutes post injection (p < 0.05). Evaluation with the paired t-test revealed no significant increase between 90 and 120 minutes (p = 0.308) or between 120 and 180 minutes (p = 0.085). However, the values at 90 and 180 minutes were significantly different (p = 0.008). Similar patterns were seen in the other compartments (data not shown).

### Comparison of relaxation time of the meniscus and femoral cartilage after contrast agent injection

**Figure 3:**
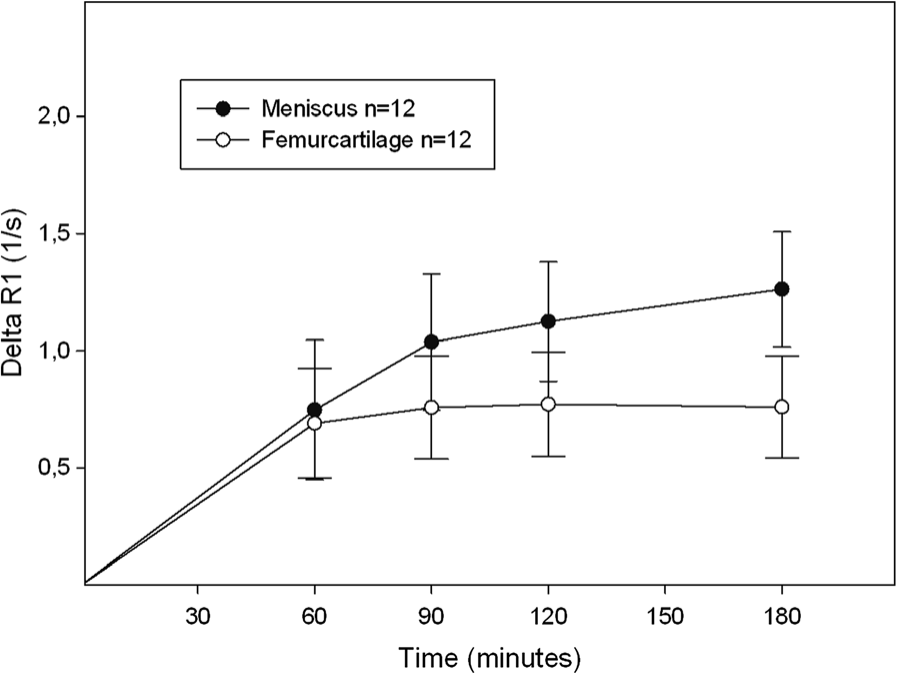
**Change in relaxation rate (ΔR**_**1**_ **± SD) in the meniscus (•) and the femoral cartilage (ο) in the posterior medial compartment after injection of a double dose of Gd-DTPA**^**2- **^**(0.2 mmol/kg body weight).** The values were significantly higher in the meniscus than in the femoral cartilage 90-180 minutes post injection (0.001 ≤ p ≤ 0.017).

The results in Figure 3 show that the change in relaxation rate in the posterior medial meniscus was generally higher than in the corresponding femoral cartilage, at all times after the double dose injection (0.001 ≤ p ≤ 0.017). Similar results were seen in the posterior lateral compartment at all times post injection (0.001 ≤ p ≤ 0.008). In the anterior compartments, a significant difference was seen in the lateral (p < 0.001) but not in the medial compartment (0.001 ≤ p ≤ 0.720). Similar patterns were observed after the triple dose injection (data not shown).

### Comparison of relaxation time of different parts of the meniscus after contrast agent injection

**Figure 4:**
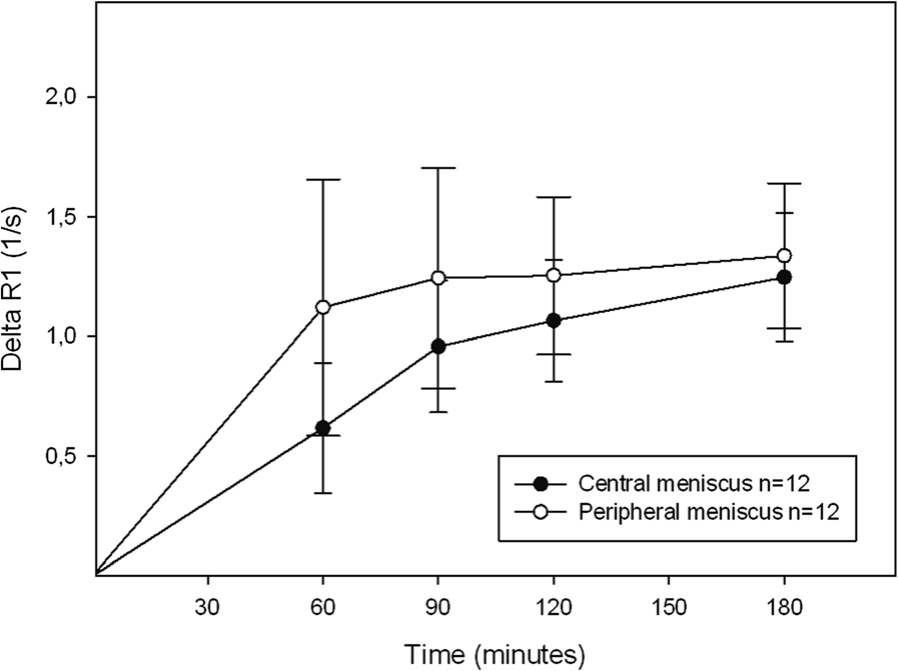
**Change in relaxation rate (ΔR**_**1**_ **± SD) in the posterior medial meniscus after injection of a double dose of Gd-DTPA**^**2- **^**(0.2 mmol/kg body weight).** The mean values over time were significantly higher in the peripheral (ο) than in the central (•) part of the meniscus (ANOVA, p = 0.024).

Comparisons of different parts of the posterior medial and lateral meniscus revealed a faster increase in ΔR_1_ (i.e. steeper curve) during the first 60 minutes in the peripheral vascular region, than in the central avascular region (p = 0.022 medial, p = 0.007 lateral). This is illustrated for the medial meniscus in Figure 4. Furthermore, the mean values of ΔR_1_ over time were significantly higher in the peripheral than in the central part of the posterior medial meniscus (p = 0.024). A difference was seen in the lateral meniscus, but it was not significant (p = 0.175).

**Figure 5:**
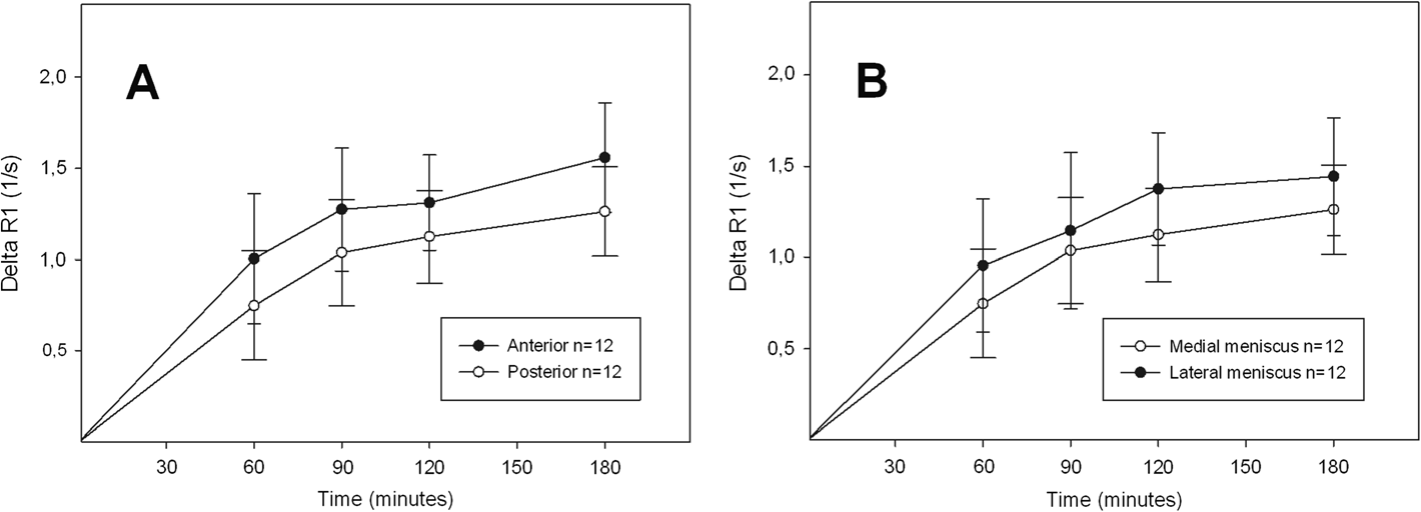
**Change in relaxation rate (ΔR**_**1**_ **± SD) reflecting the concentration of Gd-DTPA**^**2-**^**, after injection of a double dose (0.2 mmol/kg body weight) of contrast agent. A)** presents the results for the medial meniscus, showing that the mean values over time were significantly lower in the posterior (ο) than in the anterior (•) horn of the meniscus (ANOVA, p = 0.015). **B)** presents the results for the medial and lateral posterior meniscus, showing that ΔR_1_ was lower in the medial (ο) than in the lateral (•) horn (ANOVA, p = 0.049).

Comparison of ΔR_1_ in the posterior and the anterior horn of the meniscus showed a lower value in the posterior horn, reflecting a lower concentration of Gd-DTPA^2-^. Similar results were seen in the medial and lateral compartments (Figure 5A) (p = 0.015 in both compartments). ΔR_1_ was lower in the posterior medial meniscus than in the posterior lateral meniscus (Figure 5B). Similar results were seen in the anterior horn of the meniscus, (p = 0.049 and p = 0.003).

Similar results were observed within and between different parts of the meniscus in different compartments after the triple dose (data not shown).

## Discussion

The dGEMRIC method includes an injection of contrast agent and the patient also need to wait after the injection to do the MRI scan. In clinical use it would be better with a non-invasive MRI technique. T2 mapping and T1rho are such techniques. Both T2 and T1rho are thought to be sensitive to tissue hydration and matrix macromolecular architecture. T2 has been shown to be increased in subjects with pre-OA and also in subjects at risk for developing OA [30]. Reports indicate that T1rho may be more sensitive to cartilage degeneration than T2 mapping [31]. Those results are promising but further investigations are needed to evaluate the relative strengths and weaknesses of T2 and T1rho before they can be in clinical use.

In this study of contrast distribution in the meniscus and femoral joint cartilage, ΔR_1_ values, reflecting the Gd-DTPA^2-^ concentration, were considerably higher in the meniscus than in the femoral cartilage 90 to 180 minutes after injection. The triple dose yielded higher concentrations of Gd-DTPA^2-^ in the meniscus and cartilage than the double dose, but provided no additional information. The double dose of Gd-DTPA^2-^ was sufficient to detect all compartmental and regional differences, and appears to be adequate in dGEMRIC analysis of both the meniscus and cartilage. This is consistent with previous results in articular cartilage [18,32].

No statistically significant increase in ΔR_1_ was seen in the meniscus between 90 and 120 minutes or between 120 and 180 minutes. However, there was a difference between the values at 90 and 180 minutes. Thus, there was a trend of increasing ΔR_1_ after 90 minutes in the meniscus, but the increase between successive points in time was not significant. Mayerhoefer et al. suggested that 2.5-4.5 hours was a suitable time window for T_1(Gd)_ mapping of the meniscus, based on the investigation of six healthy volunteers over a period of 1-9 hours [27]. From a clinical point of view, it is advantageous to be able to examine the patient as soon as possible after injection. It is also desirable to be able to examine the cartilage and meniscus simultaneously. The results of the present study indicate that a delay of approximately 1½ hour after the injection of contrast agent is sufficient to allow uptake of Gd-DTPA^2-^ into both the cartilage and meniscus. This is in good agreement with previously published *in vivo* data [18,26].

It may be argued that an increase in the level of Gd-DTPA^2-^ in meniscus blood vessels affects the relaxation rate. The negligible volume of the blood vessels compared to the total volume of the meniscus, in combination with the fact that measurements were made up to 180 minutes post injection (the half-life of Gd-DTPA^2-^ in plasma is approximately 20 minutes), suggest that Gd-DTPA^2-^ in the blood vessels is not a source of bias [33].

The distribution of Gd-DTPA^2-^ in the cartilage, as well as different parts of the meniscus, depends on several factors that are currently not fully understood. Experimental studies on cartilage suggest that the distribution is determined to a large extent by the GAG content of the tissue [24,25]. Thus, the higher concentration of contrast agent observed in the meniscus in this study suggests that the meniscus contains less GAG than femoral cartilage. Similar results have been reported in several experimental studies of cartilage in the meniscus of dogs and rabbits [34,35]. A larger contact area with the synovial fluid, possibly facilitating the influx of contrast agent from both sides of the meniscus, may also contribute to the higher Gd-DTPA^2-^ concentration in the meniscus. Moreover, the fact that the peripheral one-third of the meniscus is vascularized probably facilitates the uptake of Gd-DTPA^2-^ by the meniscus, at least in the peripheral layer, leading to higher values than seen in the femoral cartilage. This explanation is supported by the faster increase in ΔR_1_ seen in the vascularized part of the meniscus during the first 60 minutes post injection. Other factors may also contribute to the Gd-DTPA^2-^ distribution. For example, it was recently shown that cartilage thickness strongly affects the bulk cartilage contrast agent concentration [36].

GAG content is related to differences in load distribution and weight-bearing between joint surfaces [37]. A higher content of GAG has been reported in the central zone than in the peripheral zone of the meniscus in rabbits and humans [38,39]. Due to the need for compressive integrity, cells in the central two-thirds of the meniscus synthesize more GAG than those in the peripheral one-third [40]. Thus, the differences in the distribution of contrast agent in the different compartments of the knee joint and in the different parts of the meniscus observed in this study may reflect spatial differences in GAG content caused by adaptation to biomechanical demands.

Increased GAG content, associated with early degenerative changes (Gr I or Gr II according to the Outerbridge classification), has been reported following a histochemical study using Safranin-O staining of the inner body of the meniscus in human knees [41]. Although no degenerative changes were seen in the menisci in this study, minor lesions with no symptoms, not detectable with MRI, may have been present. This may partly explain the spread in ΔR_1_ values observed in the menisci in the present study (as shown by the wide range of the 95% CIs in Figure 2, Figure 3, Figure 4 and Figure 5). A wide range of post injection T_1_ values was also reported in a previously published dGEMRIC study of the meniscus [26], while Mayerhoefer et al. did not report or discuss a wide variation in values [27]. To determine the diagnostic value of dGEMRIC in predicting meniscal degeneration consistent with early OA, the change in relaxation rate in patients with a diseased meniscus must be compared with a well-defined reference range of values obtained from healthy menisci*.*

## Conclusions

A triple dose of contrast agent yielded higher concentrations of Gd-DTPA^2-^ in the meniscus and cartilage than the double dose, but provided no additional information. It is feasible to examine undamaged meniscus and cartilage simultaneously using dGEMRIC at 90 minutes after the injection of Gd-DTPA^2-^ at a dose of 0.2 mmol/kg body weight. The uptake of contrast agent differs both within the meniscus, and in the meniscus at different locations in the knee joint.

## Competing interests

The authors declare that they have no competing interests.

## Authors’ contributions

US, CJT and LED participated in the design of the study. CS and JS developed the pulse sequence used to acquire the 3D T_1_ maps. EL and CS developed the software to enable the analyses of the MRI images. US analysed the MRI images and performed the statistical analysis. All the authors conceived the aspects of this study, and participated in its coordination. All authors read and approved the final manuscript.

## Pre-publication history

The pre-publication history for this paper can be accessed here:

http://www.biomedcentral.com/1471-2474/15/226/prepub
